# Frequent somatic mosaicism in T lymphocyte subsets in individuals with and without multiple sclerosis

**DOI:** 10.3389/fimmu.2022.993178

**Published:** 2022-12-23

**Authors:** Lies Van Horebeek, Nina Dedoncker, Bénédicte Dubois, An Goris

**Affiliations:** ^1^ Laboratory for Neuroimmunology, Department of Neurosciences, Leuven Brain Institute, Katholieke Universiteit (KU) Leuven, Leuven, Belgium; ^2^ Department of Neurology, University Hospitals Leuven, Leuven, Belgium

**Keywords:** multiple sclerosis, T lymphocytes, somatic variants, mosaicism, human genetics

## Abstract

**Background:**

Somatic variants are variations in an individual’s genome acquired after the zygotic stadium and result from mitotic errors or not (fully) repaired DNA damage.

**Objectives:**

To investigate whether somatic mosaicism in T lymphocyte subsets is enriched early in multiple sclerosis (MS).

**Methods:**

We identified somatic variants with variant allele fractions ≥1% across the whole exome in CD4^+^ and CD8^+^ T lymphocytes of 21 treatment-naive MS patients with <5 years of disease duration and 16 partially age-matched healthy controls. We investigated the known somatic *STAT3* variant p.Y640F in peripheral blood in a larger cohort of 446 MS patients and 259 controls.

**Results:**

All subjects carried 1-142 variants in CD4^+^ or CD8^+^ T lymphocytes. Variants were more common, more abundant, and increased with age in CD8^+^ T lymphocytes. Somatic variants were common in the genes *DNMT3A* and especially *STAT3*. Overall, the presence or abundance of somatic variants, including the *STAT3* p.Y640F variant, did not differ between MS patients and controls.

**Conclusions:**

Somatic variation in T lymphocyte subsets is widespread in both control individuals and MS patients. Somatic mosaicism in T lymphocyte subsets is not enriched in early MS and thus unlikely to contribute to MS risk, but future research needs to address whether a subset of variants influences disease susceptibility.

## 1 Introduction

Somatic variants are postzygotic genetic alterations that arise in a single progenitor cell, can accumulate in the soma through cell proliferation, and result in genetically distinct cells within an individual (i.e., mosaicism). Faulty DNA replication or DNA damage through exposure to internal or external mutagens can underlie the acquisition of somatic variants (1). The somatic mutation rate is thus dependent on both genetic and environmental factors.

Multiple sclerosis (MS) is a multifactorial disorder, with heritability estimated at 50% (2). The more than 230 genetic and a handful of environmental MS risk factors that have been identified are insufficient to predict who will develop MS, resulting in stochasticity in disease susceptibility (3, 4). In addition, the mechanism of action of most risk factors is unclear.

Somatic mosaicism in immune cells can underlie monogenic disorders and increase the risk for multifactorial disorders with an immune component [reviewed in (5)]. Somatic variants underlying monogenic disorders are rare at the population level and are traditionally identified starting from individuals presenting with an unexplained phenotype. Large screenings of the general population for somatic variants in whole blood uncovered widespread clonal hematopoiesis of indeterminate potential (CHIP). This consists of expanded blood cell clones in the peripheral blood carrying somatic mutations with variant allele fraction (VAF) ≥2% (meaning present in ≥4% cells) in individuals without other hematologic abnormalities (6–8). CHIP mutations occurring in hematopoietic stem cells give rise to mutated immune cells, have been shown to affect the immune system, and increase the risk of non-hematological disorders (9).

We and others have shown that somatic variants restricted to specific lymphoid lineages (mainly CD8^+^ T lymphocytes) are frequently observed in individuals with complex autoimmune disorders such as MS or rheumatoid arthritis (RA) (10–12). The limited available data on healthy individuals suggested that lineage-restricted somatic variants may be enriched in disease (11). In addition, patients with large granular lymphocytic leukemia carrying somatic gain-of-function mutations in the *STAT3* gene in CD8^+^ T cells have up to seven times higher incidence of RA than patients without these mutations (13). Together, this leads to the hypothesis that somatic mosaicism could contribute to MS susceptibility, potentially explaining stochasticity in MS susceptibility. Here, we report an exome-wide screening for somatic mosaicism in T lymphocyte subsets in control individuals and MS patients.

## 2 Materials and methods

### 2.1 Study participants and sample collection

MS patients diagnosed based on the 2017 McDonald criteria (14) and unrelated control individuals were recruited from the University Hospitals Leuven (UZ Leuven). The study has been approved by the Ethics Committee of the University Hospitals Leuven (S60222), and written informed consent was obtained from all participants. Extensive demographic and clinical data were collected through a questionnaire and medical records. Patient inclusion for the screening cohort was restricted to Caucasian treatment-naive individuals with <5 years of disease duration. Control individuals were age-matched ( ± 5-year age window) where possible. We followed up a known *STAT3* somatic variant in a larger cohort of 446 MS patients and 259 controls.

### 2.2 Screening for somatic variants

In brief, peripheral blood (PB) samples were collected and PB mononuclear cells (PBMCs) were stored in liquid nitrogen until use. PBMCs were flow-sorted into CD4^+^ and CD8^+^ T-cell subsets, and DNA from isolated cell subsets was extracted. Sample libraries for whole-exome sequencing (WES) were prepared and sequenced with the NovaSeq 6000 technology (PE100) in a single S4 flow cell (CeGaT GmbH, Tübingen, Germany). Somatic variant calling and technical filtering were performed using an updated version of our previously described pipeline (10), using the mutation callers Mutect2 (GATK version 4.2.0.00 (15) and VarScan2 (version 1.9) (16). CD4^+^ and CD8^+^ subsets were each in turn considered as the target sample, with the other subset functioning as the reference sample. Variants were annotated and analyzed with a variety of tools and algorithms. More details can be found in the **Supplementary Methods**.

### 2.3 Replication of somatic variants

A subset of 44 randomly selected somatic variants and three biologically interesting mutations in *STAT3* and *DNMT3A* was selected for replication with deep amplicon sequencing (see **Supplementary Methods**). Variants were considered as replicated if i) the correct alternate allele was identified, ii) the VAF in the target sample is higher than the VAF in the reference sample, and iii) a significant somatic *p*-value after correction for the number of variants tested (<0.001) is reported by VarScan2.

### 2.4 Droplet digital PCR

For digital droplet PCR (ddPCR), 500 ng of gDNA extracted from whole blood was cut with 10 U *Eco*RI restriction enzyme and 1x NEBuffer *Eco*RI (Bioké, Leiden, The Netherlands) in a reaction volume of 16 μl for 1 h at 37°C. Subsequently, ddPCR was performed using 50 ng of cut gDNA in a 20-μl reaction according to the standard protocol (Bio-Rad, Hercules, California, USA) using the predesigned TaqMan genotyping assay C_342265382_10 (Life Technologies, Carlsbad, California, USA). Data were analyzed with the QuantaSoft software (version 1.7.4.0917, Bio-Rad). Samples were considered positive for the alternate allele when at least two droplets reached the threshold for detection of the alternate allele.

## 3 Results

### 3.1 Somatic variants are common in CD4^+^ and CD8^+^ T lymphocytes

We analyzed the high-coverage whole-exome sequencing data of sorted CD4^+^ and CD8^+^ T lymphocytes from 16 control individuals and 21 MS patients (the median coverage across the exome had median [min, max] across all samples of 369× [218×, 475×], **Table 1**). The MS patients had recent onset of disease (<5 years, median 1.5 years) and never received disease-modifying treatment. There was no significant difference in age between cases and controls (*t*-test: *p* = 0.91). Nevertheless, a subset of 13 controls and 13 individuals with MS was age-matched even more strictly for a ± 5-year window (median difference of 0.78 years). Analyses sensitive to age were repeated for the age-matched subset, but the results did not differ substantially (see **Supplementary Data**).

**Table 1:** The study participants’ characteristics.

	CTRL (*N* = 16)	MS (*N* = 21)
Gender		
Female	7 (43.8%)	15 (71.4%)
Male	9 (56.3%)	6 (28.6%)
Age		
Mean (SD)	44.2 (14.7)	44.7 (14.2)
Median [min, max]	39.8 [28.2, 78.9]	46.1 [16.5, 65.7]
Age at onset		
Mean (SD)	–	42.7 (13.9)
Median [min, max]	–	41.4 [16.1, 61.9]
Disease duration		
Mean (SD)	–	2.05 (1.62)
Median [min, max]	–	1.50 [0.285, 4.88]
Disease course		
PPMS	–	3 (14.3%)
RRMS	–	16 (76.2%)
Unknown	–	2 (9.6%)
Smoking status		
Previous smoker	3 (18.8%)	5 (23.8%)
Current smoker	1 (6.3%)	5 (23.8%)
Never smoker	11 (68.8%)	11 (52.4%)
Unknown	1 (6.3%)	0 (0%)

Somatic variants acquired by mature T cells were called using an updated version of our previously published pipeline (10). We identified 878 somatic variants with high confidence (**Supplementary Table 4**) and selected 47 variants for replication through deep amplicon sequencing. We obtained high coverage sequencing data from both the CD4^+^ and CD8^+^ subsets for 33 variants (median [min, max] coverage across samples of 10,449× [590×, 50,932×]) and replicated 29 variants (replication rate of 87.9%), confirming the high confidence in the somatic variants identified.

**Figure 1:**
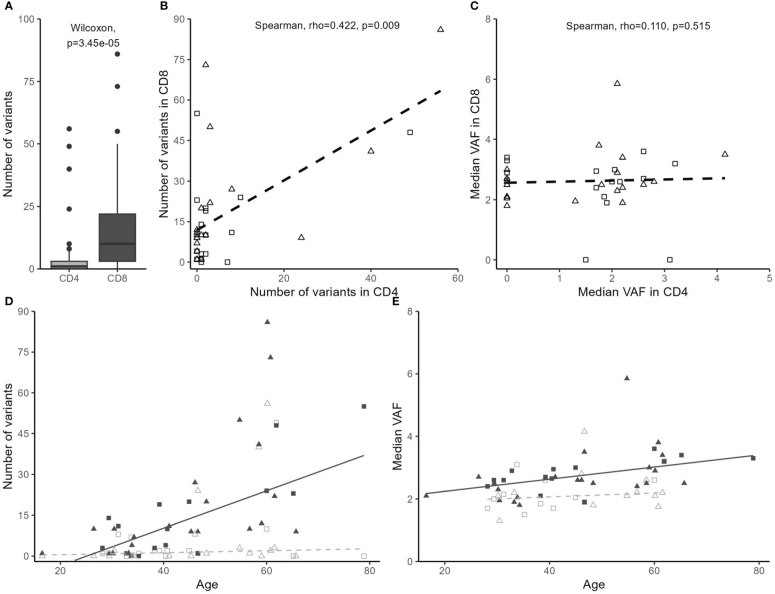
Somatic variants are more common in CD8^+^ than in CD4^+^ T lymphocytes and are correlated with the individual’s age. Data points from controls are depicted by squares and from individuals with MS by triangles. **(D, E)** Data points from the CD4^+^ subset are depicted in light gray and without fill and from the CD8^+^ subset in solid dark gray. Linear regression trend lines are shown. **(A)** Distribution of the number of somatic variants in CD4^+^ and CD8^+^ subsets. **(B)** Correlation between the number of somatic variants in CD4^+^ and CD8^+^ subsets. **(C)** Correlation between median variant allele frequency (VAF) of somatic variants in CD4^+^ and CD8^+^ subsets, with median VAF set to 0 in absence of somatic variants. **(D)** The number of variants in CD4^+^ and CD8^+^ T lymphocytes in correlation with the individual’s age [quantile (median) linear regression: the number of variants differs with cell type (*p* = 0.031) and significantly increases with age in the CD8^+^ subset (*p* = 0.001), but not in the CD4^+^ subset (*p* = 0.697)]. **(E)** Median VAF of variants in CD4^+^ and CD8^+^ T lymphocytes across individuals in correlation with the individual’s age [quantile (median) linear regression: median VAF estimated to be 0.68% higher in the CD8^+^ subset (*p* = 2.9 × 10^−4^) and to increase with 0.02% per 1-year increase in age (*p* = 0.018).

The number of high-confidence somatic variants identified per individual varied greatly (1-142 variants per individual; **Figure 1A**), and this number was not correlated with the sequencing depth of either the target or the reference sample (not shown). The number of somatic variants in CD4^+^ and CD8^+^ T lymphocytes of the same individual was correlated (Spearman rho = 0.422, *p* = 0.009), but not their median VAF (**Figures 1B, C**). Somatic variants were most abundant in the CD8^+^ subset (Wilcoxon: *p* = 3.45 × 10^−5^ versus CD4^+^, **Figure 1A**) and increased with age in CD8^+^ (linear regression: *p* = 0.001), but not in the CD4^+^ subset (*p* = 0.697) (**Figure 1D**). Median VAF was estimated to be 0.68% higher in CD8^+^ than in CD4^+^ (*p* = 2.9 × 10^−4^) and increased with 0.02% per 1-year increase in age (*p* = 0.018) (**Figure 1E**). Neither the number of variants nor the median VAF was associated with smoking status (*p* > 0.30).

### 3.2 Somatic variants are not associated with MS disease status

**Figure 2:**
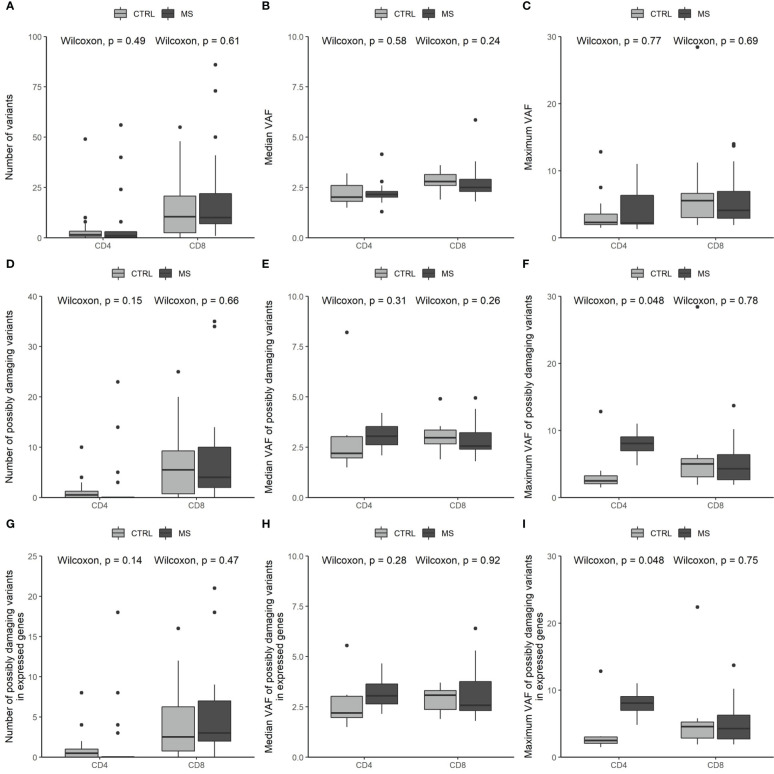
Somatic variants are similar in number and abundance in control individuals and MS patients. **(A–C)** Number **(A)**, median VAF **(B)**, and maximum VAF **(C)** of somatic variants in CD4^+^ and CD8^+^ T lymphocytes in control individuals and individuals with MS. **(D–F)** Number **(D)**, median VAF **(E)**, and maximum VAF **(F)** of somatic variants predicted to be damaging in CD4^+^ and CD8^+^ T lymphocytes in control individuals and individuals with MS. **(G–I)** Number **(G)**, median VAF **(H)**, and maximum VAF **(I)** of somatic variants predicted to be damaging and expressed in the affected cell subset in CD4^+^ and CD8^+^ T lymphocytes in control individuals and individuals with MS.

We compared somatic mosaicism between individuals with and without MS. We identified similar numbers and VAFs of high-confidence somatic variants in control individuals and MS patients (**Figures 2A–C**). We subsequently restricted the analysis to possibly damaging variants (**Figures 2D–F**), i.e., variants that i) were non-synonymous or affecting translation start or stop sites, ii) were located in genes not frequently mutated (with germline variants) in the healthy population (GDI < 13.84), and iii) were predicted to be damaging (CADD score ≥ 20). Finally, we analyzed the possibly damaging variants in genes that are known to be expressed in the relevant T-cell subset (CD4^+^ or CD8^+^) based on public databases, so that they have the potential to exert an effect (**Figures 2G–I**). Overall, the possibly damaging variants, either all or those in expressed genes, did not occur more often or more abundantly in individuals with MS. The CD4^+^ T cells of MS patients showed a trend toward a higher maximum VAF, corresponding to a possibly larger clonal size, both for all possibly damaging variants and for possibly damaging variants in expressed genes (*p* = 0.048, **Figures 2F, I**), although this does not survive multiple testing correction.

### 3.3 Somatic variants have characteristics indicating deleteriousness but are enriched in lowly expressed genes

**Figure 3:**
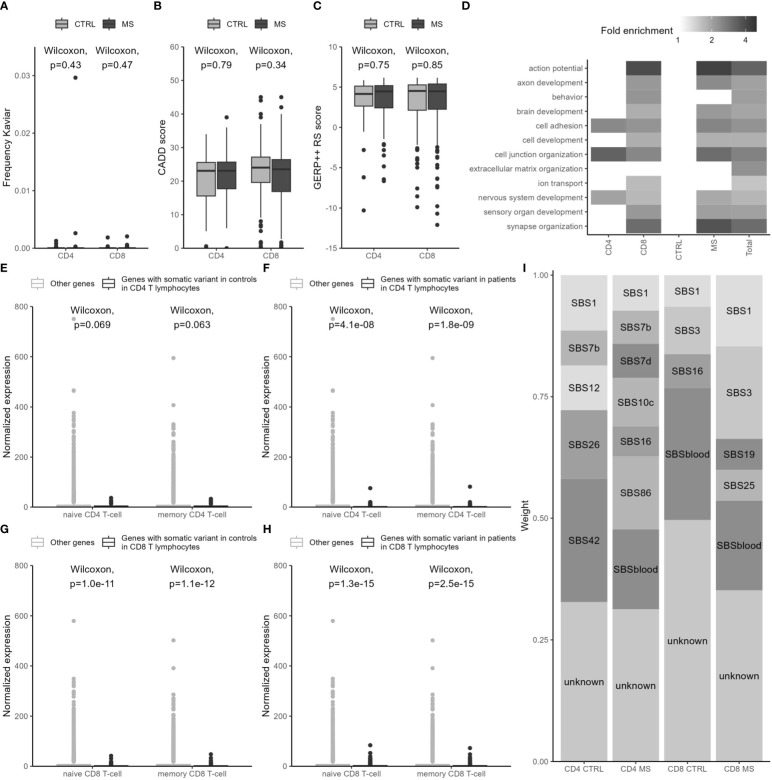
Somatic variants have characteristics compatible with a damaging effect but are located in lowly expressed genes not specific for the affected cell subsets. **(A)** Frequency of somatic variants in Kaviar, a database for germline variants. **(B)** CADD scores of somatic variants as an estimate of predicted deleteriousness. **(C)** GERP++ score as a measure of conservation of sites at which somatic variants are located. **(D)** Fold enrichment of a subset of GO terms related to biological processes in different sets of somatic variants. As fewer controls were included, the power of this analysis was insufficient to obtain results with a false discovery rate (FDR) ≤5%. Fold enrichment is only included for over- and underrepresentations with an FDR ≤5%. An overview of all GO terms with FDR ≤5% is given in **Supplementary Figure 3**. **(E–H)** Normalized gene expression in the relevant CD4^+^ and CD8^+^ T-cell subsets for genes affected by somatic variants in CD4^+^ in controls **(E)**, in CD4^+^ in MS patients **(F)**, in CD8^+^ in controls **(G)**, and in CD8^+^ in MS patients **(H)** and other genes screened for somatic variants. **(I)** Mutational signatures, consisting of predefined combinations of single base substitutions (SBS), found in sets of somatic variants.

Our dataset obtained from investigating the whole exome allows unbiased evaluation of somatic variant characteristics. Overall, somatic variants were not often observed as germline variants in the public database Kaviar: the majority was predicted to be damaging according to germline variant effect predictors (CADD score ≥ 20), and most somatic variants were located at conserved positions (GERP++ score > 3) (**Figures 3A–C**). However, genes affected by somatic variants were not highly expressed in the cell types in which the variants were identified but were instead highly significantly enriched for low T-cell expression levels (**Figures 3E–H**) and were not specific for T cells or T-cell subsets (**Supplementary Figure 1**). The frequency in Kaviar, CADD score, and GERP score did not significantly differ between variants in expressed and non-expressed genes (**Supplementary Figure 2**). The affected genes were overrepresented for GO terms related to the (central) nervous system, ion transport, and developmental processes, but not for GO terms related to the immune system or cell proliferation (**Figure 3D** and **Supplementary Figure 3**). A subset of 62 somatic variants (7.0%) overlapped with the Catalogue of Somatic Mutations in Cancer (COSMIC), with four (0.46% of all variants) in hematopoietic and lymphoid tissues. None of the characteristics differed between CD4^+^ and CD8^+^ T cells or between MS patients and controls.

### 3.4 Mutational signatures suggest differences in underlying mutational processes between CD4^+^ and CD8^+^ T lymphocytes

As individual mutagenic exposures induce mutations through specific mechanisms, each type of exposure leads to a characteristic combination of mutation types, also called a mutational signature. We used the mutational signatures identified in cancer and in normal hematopoietic cells to decipher relevant mutational processes (**Figure 3I**). Age-related signature 1 was identified across cell types and disease status. Interestingly, UV-related signatures (SBS7b/d) were only identified in CD4^+^ subsets, whereas DNA repair-related signature 3 was unique to the CD8^+^ subsets. SBSblood was identified in the three largest sets of variants (not in CD4^+^ in controls).

### *3.5 STAT3* and *DNMT3A* are hotspots for somatic variants

Frequently occurring variants or regions frequently mutating—so-called hotspots—may indicate mutations leading to a survival or proliferation advantage. Within this screening, no identical somatic variants were observed in multiple individuals, but 56 genes carried more than one somatic variant (2-5 variants per gene, 123 variants in total). We identified two known CHIP driver mutations in *DNMT3A* (17), p.R483W and p.R597C (both confirmed experimentally), in CD8^+^ T lymphocytes of different MS patients with a VAF of 2.6%-2.8%. No CHIP driver mutations in *DNMT3A* affecting both CD4^+^ and CD8^+^ subsets could be identified. Across our study and others, *STAT3* and the *STAT3* pathway were frequently mutated (11, 12, 18). We identified *STAT3* p.Y640F in CD8^+^ T lymphocytes of an individual with MS with a VAF of 3.3% (confirmed experimentally). To explore the abundance and disease specificity of the STAT3 p.Y640F mutation, we screened whole blood genomic DNA from 259 healthy controls and 446 MS patients using the sensitive ddPCR (**Table 2**, **Supplementary Table 1**, **Supplementary Figure 4**). We first applied ddPCR to the patient from the screening cohort in which the *STAT3* p.Y640F variant was detected in CD8^+^ T cells. The VAF of 2.58% in CD8^+^ T cells estimated by ddPCR is close to the 3.3% estimated by sequencing in the screening phase. The absence of any droplet positive for the alternate allele in triplicate measurements of the negative CD4^+^ T cells (by deep sequencing determined) suggests a low false positive rate for this assay. The false positive rate was reduced even further by requiring at least two positive droplets to be considered positive. The variant was also detectable in the whole blood genomic DNA of the patient, with VAF 0.068%-0.16% across four different time points spanning 4 years. Upon screening whole blood genomic DNA in the larger cohort, the variant allele could be detected confidently in 4.2% of healthy controls and 4.0% of MS patients (logistic regression, corrected for age: *p* = 0.973). In those individuals, the VAF was not significantly different between healthy controls and MS patients (linear regression, corrected for age: *p* = 0.231).

**Table 2:** Detection of *STAT3* p.Y640F somatic variant in the peripheral blood of MS patients and controls.

	MS (*N* = 446)	CTRL (*N* = 259)	p*
Age at sampling			2.84 × 10^−04^
Mean (SD)	44.1 (13.1)	48.0 (13.9)	
Median [min, max]	44.0 [16.0, 78.0]	48.0 [19.0, 82.0]	
Missing	0 (0%)	3 (1.2%)	
Disease duration at sampling			
Mean (SD)	10.6 (9.35)	–	
Median [min, max]	7.00 [0, 52.0]	–	
Missing	4 (0.9%)	–	
Presence of an alternate allele			0.973
Yes	18 (4.0%)	11 (4.2%)	
No	428 (96.0%)	248 (95.8%)	
VAF in individuals carrying the alternate allele			0.231
Mean (SD)	0.072% (0.057%)	0.050% (0.037%)	
Median [min, max]	0.047% [0.014%, 0.18%]	0.041% [0.018%, 0.16%]	

*Wilcoxon test for the age difference, logistic regression with age as a covariable for the presence of an alternate allele and linear regression with age as a covariable for VAF in individuals carrying the alternate allele.

## 4 Discussion

We previously developed a pipeline to identify somatic variants with low VAFs in a non-cancer context and demonstrated as a proof of principle that somatic variants in immune cells are common in autoimmune diseases (10). We found that variants detected in T lymphocytes were restricted to either the CD4^+^ or the CD8^+^ subset (10). We now characterized somatic variants directly in T lymphocyte subsets in individuals with and without MS and investigated their enrichment in early disease using this pipeline in combination with deep whole-exome sequencing.

We report a widespread presence of somatic mosaicism across the exome of T lymphocyte subsets, in line with other recent studies using gene panels (18–20). As we strived to achieve a high true positive rate, we expect that we underestimate the true number of somatic variants present in the exome. Our findings confirm that somatic variants are more common and more abundant in CD8^+^ cells compared with CD4^+^ cells (10–12, 20), and this difference becomes even more pronounced with age. The observed differences may be a consequence of more clonal expansion after activation of CD8^+^ cells and larger CD8^+^ clones, compared with CD4^+^ (21, 22). Indeed, clonality based on T-cell receptor (TCR) sequencing is higher in the CD8^+^ subset and correlated with mutation burden (19, 20). TCR-based clonality of CD8^+^ and CD4^+^ was partially correlated (19, 20), in line with our observation of correlated mutational burden. Differences in underlying mutational processes between cell types, as suggested by our observation of a subset of mutational signatures present in only one subtype, may also contribute to increased mutational load in CD8^+^ cells. In line with other studies (19, 20, 23), we see a consistent role for the age-related mutational signature SBS1 and the hematopoietic stem and progenitor cell endogenous mutational signature SBSblood. The homologous recombination-based repair signature 3, observed in CD8^+^ cells, is also associated with indels and larger genome rearrangement signatures, pointing to the relevance of also investigating structural somatic mosaicism in the future.

Our whole-exome approach enabled us to characterize somatic variants in an unbiased way, in contrast to the few similar studies based on gene panels enriched for immune genes (11, 12, 18–20). Whereas somatic variants have predicted characteristics indicating an effect on gene function, most affected genes seem to be irrelevant to T-cell functioning. This is in line with previous observations of a negative correlation between expression levels and the number of mutations both in cancer and in healthy tissues, likely at least partially explained by the transcription-coupled repair mechanisms (24, 25). We currently do not know whether the few somatic variants in expressed genes are neutral and hence tolerated or whether some of them alter the function of the gene, the cell, and the immune system. Future studies should try to make a distinction between driver and passenger mutations, similar to the cancer field.

The inclusion of both controls and individuals with MS with recent disease onset (<5 years) and who were never treated allowed us to evaluate whether somatic variants are specific to or enriched in disease. The power to detect differences was limited, due to the detection of somatic mosaicism in all individuals and the large variability within groups. We estimate >80% power to detect 2–4-fold differences in the number of somatic variants and >1.5-fold differences in median VAF. In this cohort, we do not see differences with regard to disease status in either the CD4^+^ or CD8^+^ subset, with the exception of a trend toward higher maximal VAF, and hence potentially higher clonality, for possibly damaging variants in CD4^+^ T cells of MS patients. Our findings correspond to the findings from another research group investigating CD8^+^ T lymphocytes only, who did not observe a significant difference in the number of somatic variants in 2,524 immunity and cancer-related genes between 21 recently diagnosed individuals with MS and 21 sex- and age-matched controls (18). Similarly, no significant difference was observed for other immune-mediated diseases such as primary immunodeficiency, often associated with autoimmunity (19). In contrast, enrichment was observed in CD8^+^ T cells in aplastic anemia, although at least a partial effect of age cannot be excluded (20). The lack of enrichment in early disease, combined with the relative stability of somatic variants in immune cells over up to 5 years or more (5, 10, 12), suggests a lack of enrichment at and before the time of diagnosis. Therefore, it is unlikely that the mere absence or presence of somatic mosaicism in T lymphocyte subsets i) explains stochasticity in disease susceptibility or ii) provides a mechanism of action for increased risk from germline or environmental risk factors.

Similar to germline variants, only a subset of somatic variants may influence disease susceptibility. Identification of an MS-relevant subset is even more challenging for somatic variants due to the larger variety of variants and the additional dimensions of time (when did the variant occur) and space (which cell subsets are affected). It would be reasonable to assume that somatic variants affecting disease must first alter normal gene and cell function, but this information is likely only occasionally available. An alternative approach to identify the relevant subset is through hotspots, i.e., recurrent variants or variants recurring at specific genomic regions, for which much larger datasets are desirable. Combining our dataset with other studies on MS and immune-mediated disorders, we notice multiple occurrences in the *STAT3* pathway (11, 12, 18, 20). *STAT3* is a known MS risk gene (26), and somatic gain-of-function mutations in this gene in CD8^+^ T cells in large granular lymphocytic leukemia are associated with an increased incidence of RA (13). We thus screened a larger cohort of MS patients and controls for the *STAT3* p.Y640F gain-of-function variant (27). The *STAT3* variant was detected in ~4% of individuals in both groups. This is in line with another MS study observing more *STAT3* mutations in controls than cases and detecting the p.Y640F variant in 1/21 (4.8%) controls (18). Our data confirm that STAT3 mutations are not enriched in MS and suggest that the *STAT3* hotspot, originally identified in cancer, is not disease-specific. Cohorts of an order of magnitude larger are required to confidently determine whether this variant influences the risk for MS with a smaller effect size. In particular, this mutation presenting in one larger clone or hitting an autoreactive T cell may impact the immune and disease phenotype (20, 28). We also observed two CHIP driver mutations (17) in *DNMT3A* in CD8^+^ T lymphocytes of MS patients. Recently, CHIP—with the most common mutations in *DNMT3A* (29.5%)—was associated with an increased risk for autoimmune diseases (OR 6.6, 95% CI 1.7–30) in patients undergoing hip arthroplasty (29). So far, no studies have investigated the link between CHIP and MS. When doing so in the future in the context of MS, it may be particularly relevant to consider lymphoid-associated CHIP mutations in addition to the more widely studied myeloid-associated CHIP mutations (30). Similar to germline variants (31), somatic variants may increase the risk of autoimmunity in general or predispose toward specific autoimmune disorders.

The study design, aimed at confident variant calling, also comes with limitations. By comparing data from different T lymphocyte subsets, variants acquired before lineage commitment, and present in both subsets, are excluded. Clonal expansion of relevant clones may be more easily detected during active disease or within the CSF. Our exome-wide approach allowed unbiased comparison between individuals with and without MS but limited the sample size of our study. Targeted approaches, for example focusing on genes associated with lymphoid clonal hematopoiesis (30), will allow the inclusion of other cell types playing a role in the development of MS, cells from other compartments such as the CSF, and more individuals in future efforts.

In summary, we found that somatic mosaicism is common in T lymphocytes and mainly in CD8^+^ T lymphocytes. This fits into a context where somatic mosaicism is increasingly recognized as ubiquitous. One extreme is the realization that monozygotic twins are not fully genetically identical, but differ in on average 14 postzygotic somatic variants present in whole blood, a number that increases with age (32). Somatic mosaicism in itself does not appear to be disease-specific, but given that somatic variants in immune cells are so widespread, future research needs to evaluate whether individual somatic variants affect disease.

## Data availability statement

Summary statistics for all variants are available through the Lead Contact, Prof. An Goris (an.goris@kuleuven.be). Requests for individual-level genotype or sequence data should be directed to and will be fulfilled pending Institutional Review Board approval and accordance with EU General Data Protection Regulation by the Lead Contact, Prof. An Goris (an.goris@kuleuven.be).

## Ethics statement

The studies involving human participants were reviewed and approved by Ethics Committee of the University Hospitals Leuven. The patients/participants provided their written informed consent to participate in this study.

## Author contributions

LVH and AG: conception and design. BD: sample and data collection. LVH and ND: sample processing, experiments, and data generation. LVH: data analysis and writing of the first draft of the article. LVH, BD, and AG: data interpretation. AG: study supervision. All authors contributed to the article and approved the submitted version.
