# Supplementary material for: Frequent somatic mosaicism in T lymphocyte subsets in individuals with and without multiple sclerosis
Source: Front Immunol. 2022 Dec 23;13:993178. doi: 10.3389/fimmu.2022.993178 (PMC9817019; doi:10.3389/fimmu.2022.993178)
Supplement: Supplementary file 1 [file DataSheet_1.docx]

Supplementary Material

# Supplementary tables

**Supplementary table 1. Cohort description and results from ddPCR**

|  | **MS (N=446)** | **CTRL (N=259)** | **P value** |
| --- | --- | --- | --- |
| **Number of droplets** | | | < 2.2e-16 |
| Mean (SD) | 15000 (1880) | 13900 (2350) |  |
| Median [Min, Max] | 15300 [7490, 19200] | 14200 [5420, 17700] |  |
| **Concentration ref allele** | | | 5.563e-05 |
| Mean (SD) | 643 (315) | 571 (252) |  |
| Median [Min, Max] | 593 [20.1, 3870] | 549 [27.3, 2640] |  |
| **VAF** |  |  | 0.8993 |
| Mean (SD) | 0.0000291 (0.000180) | 0.0000213 (0.000125) |  |
| Median [Min, Max] | 0 [0, 0.00183] | 0 [0, 0.00156] |  |

All tests are Wilcoxon.

**Supplementary table 2. Regions selected for replication.**

See supplementary Excel file

**Supplementary table 3. Gene lists used in PANTHER analyses**

See supplementary Excel file

**Supplementary table 4. List of identified somatic variants**

See supplementary Excel file

# Supplementary figures


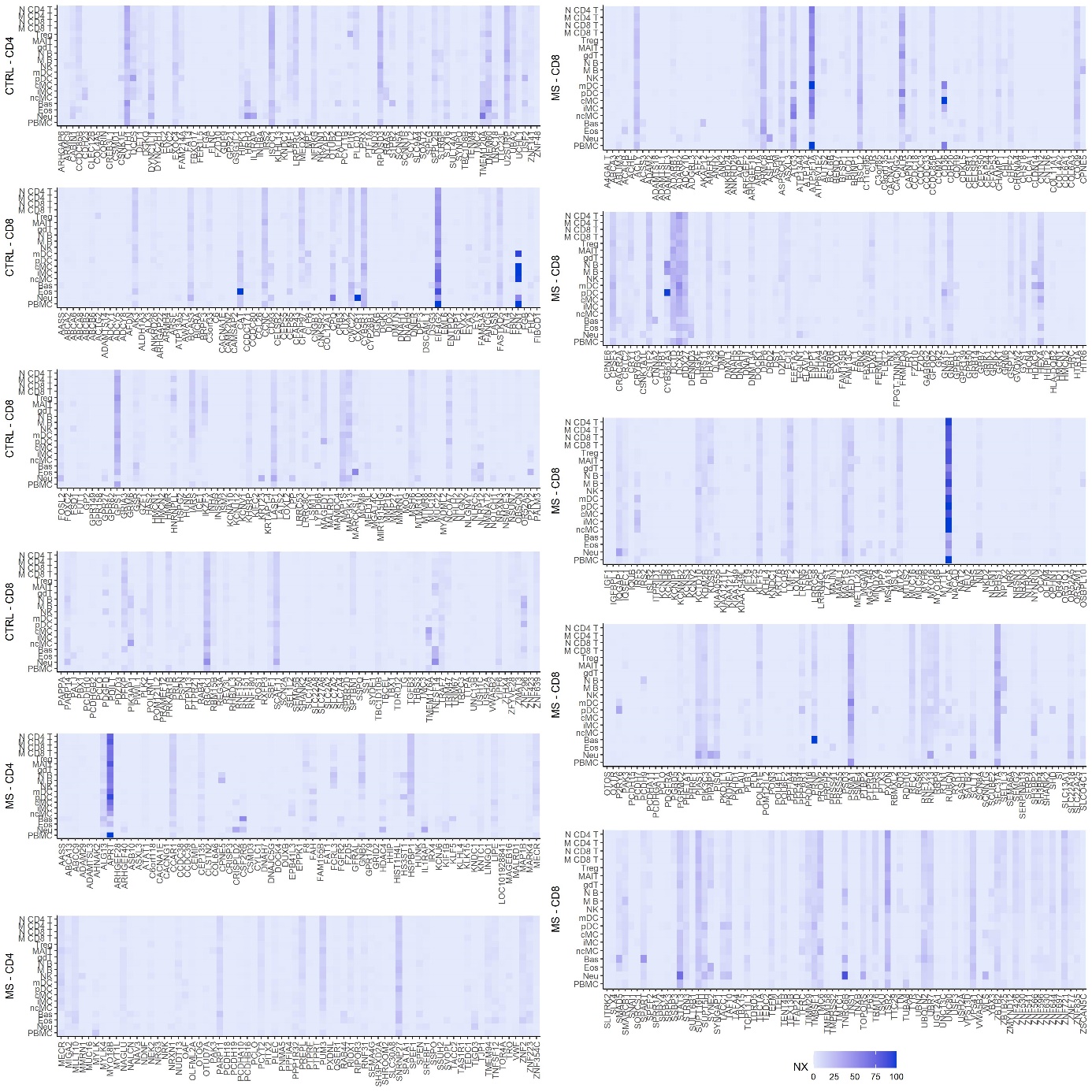


**Supplementary figure 1. Genes affected by somatic variants do not show expression specific to T lymphocytes or T lymphocyte subsets.**

Genes are grouped by cell subset and disease status and listed alphabetically. Normalized expression (NX) is capped off at 100, with no difference in color for values above this cutoff. The first seven rows are naïve CD4^+^ T lymphocytes, memory CD4^+^ T lymphocytes, naïve CD8^+^ T lymphocytes, memory CD8^+^ T lymphocytes, regulatory T lymphocytes, mucosal‑associated invariant T lymphocytes and gamma‑delta T lymphocytes.


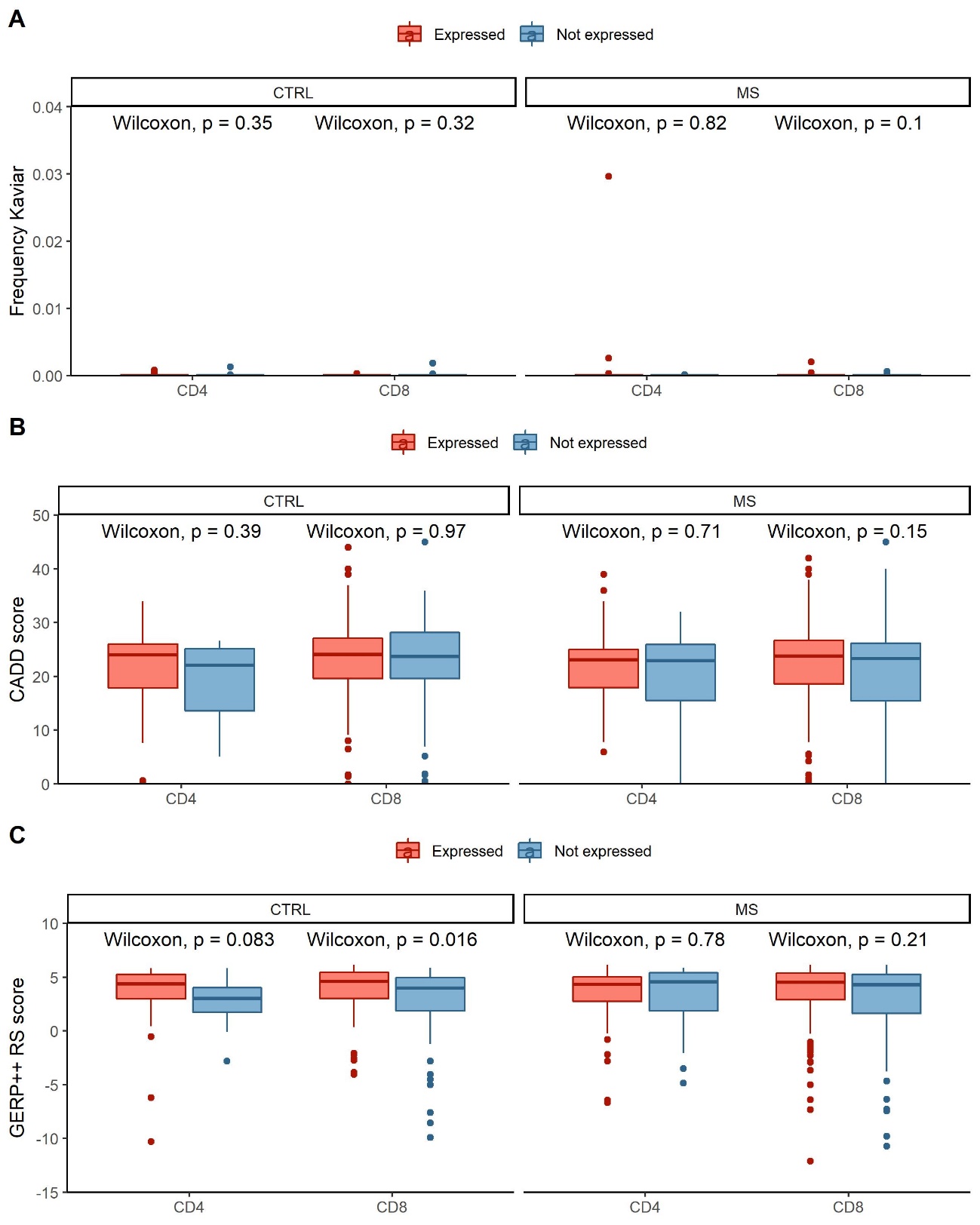


**Supplementary figure 2. Variant characteristics do not differ between expressed and non‑expressed genes.**

Gene expression is evaluated in the affected T lymphocyte subset (CD4^+^ or CD8^+^) and a gene is considered expressed if normalized expression in either naïve or memory subsets is larger than 0. **A**. Frequency of somatic variants in Kaviar, a database for germline variants. **B**. CADD scores of somatic variants as an estimate of predicted deleteriousness. **C**. GERP++ score as a measure of conservation of sites at which somatic variants are located.


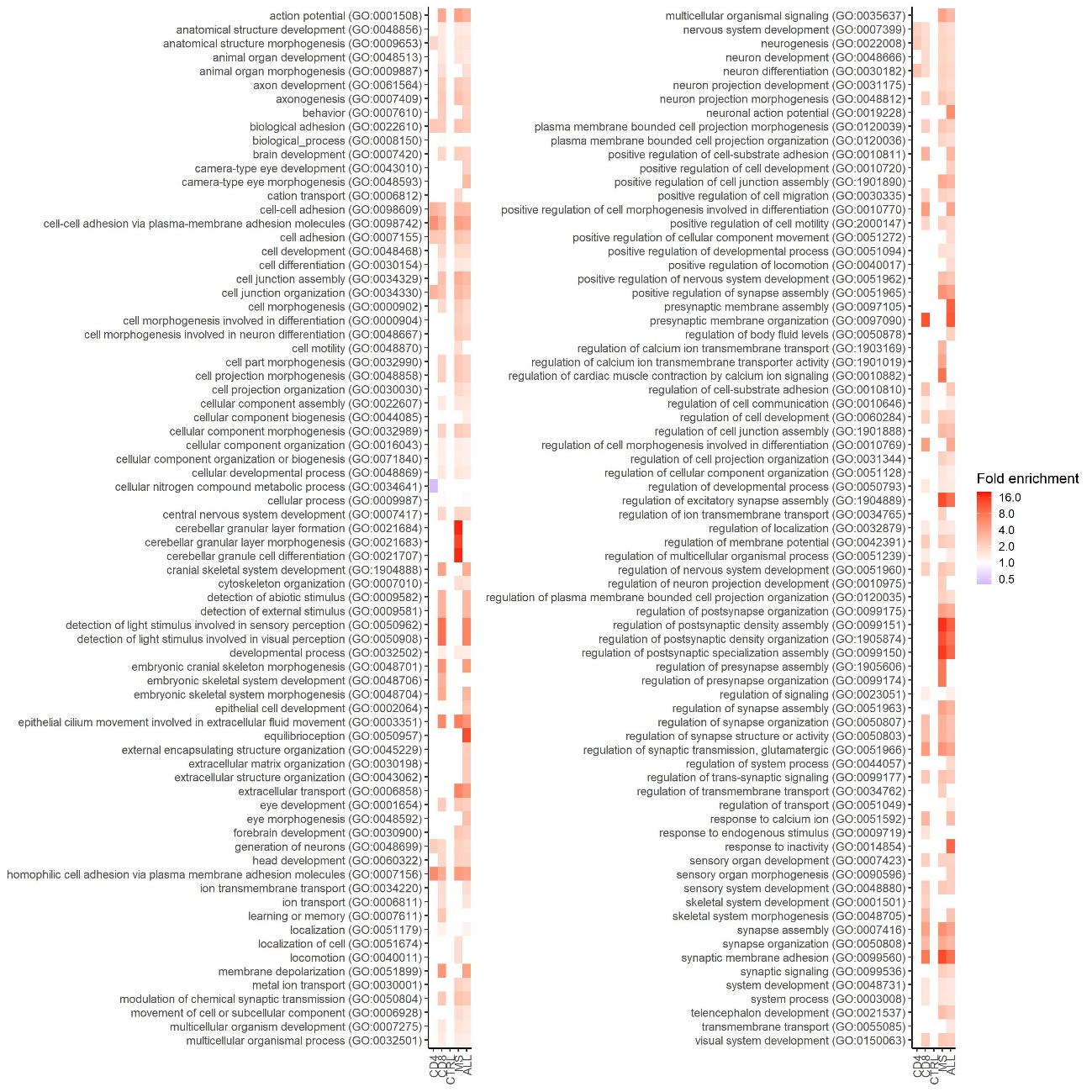


**Supplementary figure 3. GO terms related to the immune system or cell proliferation are not overrepresented in any of the subsets.**

All GO terms reaching a FDR less than 5% in any of the analyses are included in the heatmap. Fold enrichment is only indicated when FDR less than 5% is reached.


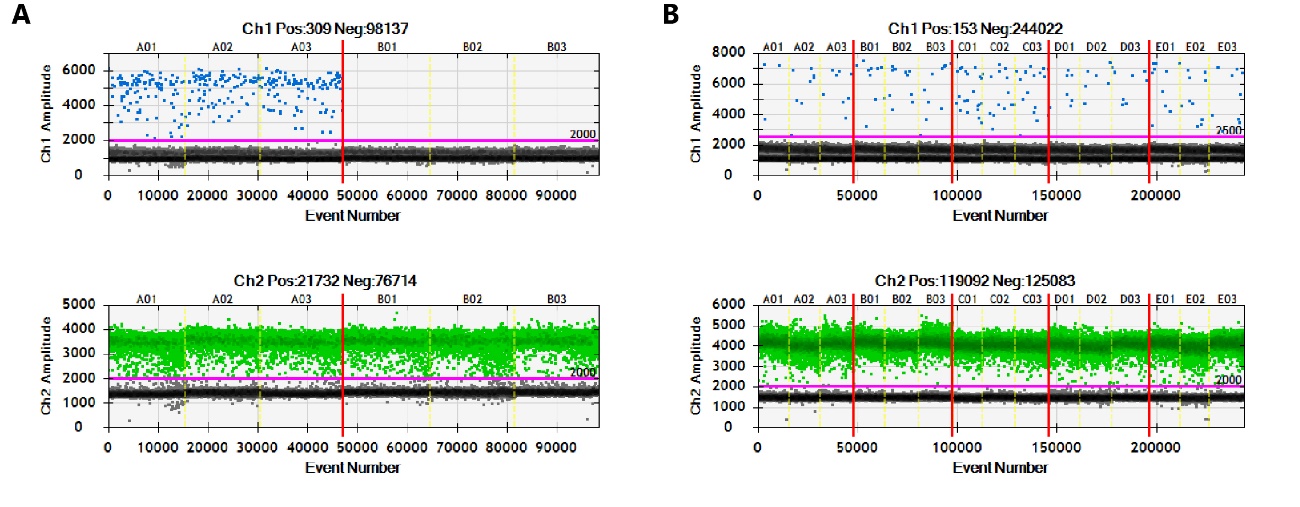


**Supplementary figure 4. Examples of the raw ddPCR data.**

Figure representing the number of droplets in a total volume of 20 µl positive for the alternate allele (blue dots) and the reference allele (green dots) in DNA obtained from the MS patient in which the somatic *STAT3* p.Y640F variant was originally identified**.** Negative droplets are indicated in grey, the threshold for positive droplets is indicated in pink, and the different samples are separated by a red line. **A.** ddPCR data from remaining DNA from the screening phase of CD8^+^ (left, positive control, VAF=2.58%) and CD4^+^ (right, negative control) subsets. **B.** ddPCR data from whole blood gDNA from five different time points, shown chronologically from left to right. The first time point overlaps with the screening phase and VAF is estimated at 0.068%. Subsequent time points are all approximately one year apart and have VAFs of 0.094%, 0.161%, 0.072% and 0.071%.

# Supplementary methods

## Screening for somatic variants

### FACS

PBMCs were collected and processed as described previously (1) and stored in liquid nitrogen until use. The following antibodies were used for staining of cells (1): FITC‑labeled anti‑human CD3 (1:50, eBioscience), eFluor® 450-labeled anti-human CD4 (1:50, eBioscience), PE-cyanine 7-labeled anti-human CD8a (1:100, eBioscience), PerCP-Cyanine5.5-labeled anti-human CD19 (1:500, eBioscience). Dead cells were stained using the LIVE/DEAD Fixable Red Dead Cell Stain Kit (Thermo Fisher Scientific), following manufacturer’s instructions. Flow cytometry was performed with a Sony MA900 instrument (Sony Biotechnology).

### Sequencing

DNA from sorted cells was extracted using an in-house extraction protocol(1). From 50 ng DNA obtained from isolated CD4^+^ and CD8^+^ T lymphocytes, sample libraries for whole exome sequencing (WES) were prepared using the Twist Human Core, Refseq and Mitochondrial Panels (Twist Bioscience). Libraries were pooled and sequenced with the NovaSeq 6000 technology (PE100) in a single S4 flow cell (CeGaT GmbH).

### Demultiplexing and mapping

Samples were demultiplexed using Illumina’s bcl2fastq (version 2.20) and adapters were trimmed with Skewer (version 0.2.2). Sequence reads were aligned to the human Reference Genome Build (NCBI37/hg19) by BWA software (version 0.7.17). Local realignment around indels and base recalibration were done using GATK (version 3.5). Duplicate reads were flagged using Picard software. Coverage was calculated using SAMtools (version 1.9), while excluding reads marked as duplicates.

**Supplementary table 5. Sequencing depth across samples**

|  | **CTRL (N=16)** | **MS (N=21)** |
| --- | --- | --- |
| **Median coverage across exome in CD4^+^ subset** | | |
| Mean (SD) | 361 (50.3) | 386 (47.8) |
| Median [Min, Max] | 371 [218, 411] | 378 [283, 475] |
| **Median coverage across exome in CD8^+^ subset** | | |
| Mean (SD) | 338 (49.6) | 346 (53.0) |
| Median [Min, Max] | 331 [260, 421] | 343 [222, 437] |
| **Percentage of exome with coverage ≥150x in both CD4^+^ and CD8^+^ subset** | |  |
| Mean (SD) | 95.4 (3.84) | 96.4 (3.37) |
| Median [Min, Max] | 97.0 [83.4, 98.6] | 97.6 [83.8, 98.6] |

### Mutect2

Mutect2 (GATK version 4.2.0.0) was run following the tools recommendations (2). Mutect2 was first run in the tumor-only mode and the output was combined into a cell-type specific “panel of normals" (CD4^+^ or CD8^+^). Subsequently, Mutect2 was run comparing the target and reference sample from the same individual, where CD4^+^ and CD8^+^ subsets were each in turn considered as target sample, with the other subset functioning as reference sample. The panel of normals of the reference cell-type and population allele frequencies from gnomAD (version 2.1.1) were used as additional input. For the additional analysis identifying somatic variants present in both CD4^+^ and CD8^+^ subset, we removed the filter on VAF in reference sample and excluded Mutect2’s normal artifact filter.

### VarScan2

SAMtools mpileup (version 1.9) was used to create pileup files per sample. When running mpileup, we disabled maximum depth and base quality adjustments and excluded reads marked as duplicates. VarScan2 (version 2.3.9) was then used for a direct comparison of target and reference sample from the same individual (3). To obtain information on all deviations from the reference genome, we lowered the minimal variant frequency to 0, minimum coverage to 1x and minimum number of reads supporting the alternate allele to 1. We compensated these settings during the later filtering steps, which leads to the same result as more stringent settings immediately.

### Background info

In order to better distinguish true somatic variants from systematic errors, which occur in many samples, we pooled information on the occurrence of the variant in reference samples in line with a previously published methodology (4). Based on the output from VarScan2, we calculated the following parameters:

- VAF_background_: fraction of all reads covering the position of the variant that support alternate allele across all reference samples;
- N_background_: number of reference samples with two or more reads supporting alternate allele;
- ratio_background_: ratio between VAF of the variant in target sample (= VAF_target_) and VAF of the variant in all reference samples (= VAF_background_).

### Selection/filtering of somatic variants

Processing of the output files and filtering of the candidate somatic variants was done in R (version 4.0.5). VCF files were loaded and converted to data frames using the R package vcfR (version 1.12.0). R packages *data.table*, *dplyr*, *splitstackshape*, *stringr* and *tidyr* were used for further data wrangling. Overlap between candidate variants found by Mutect2 and VarScan2 was based on an exact match of chromosome, position, reference allele and alternate allele. Variants were annotated using Annovar (5) with information from the following databases: refGene, Kaviar (version 20150923), ExAC03, ESP6500 (version esp6500siv2), gnomAD (version 2.1.1), 1000g (version 2015aug), COSMIC70, dbSNFP41a, ClinVar (20210501), HRCR1 and ICGC (version icgc28).

Somatic variants considered as highly confident fulfilled the following technical criteria:

- identified by both Mutect2 and VarScan2;
- passing all Mutect2 filters;
- annotated as located in an exon, a splicing region or an UTR;
- located outside segmental duplications;
- coverage according to both Mutect2 and VarScan2 in target and reference sample at least 150x;
- VAF according to both Mutect2 and VarScan2 in target sample at least 1%;
- VAF according to both Mutect2 and VarScan2 in reference sample of the same individual at most 0.5%;
- VAF_background_ at most 0.1%, N_background_ at most 2 and ratio_background_ at least 50.

High confidence somatic variants considered as potentially damaging additionally fulfilled all following criteria:

- the variant induces an amino acid change or creates or removes a start or stop site;
- the affected gene is not frequently mutated (germline) in the healthy population: Gene Damage Index (GDI) < 13.84 (6);
- the variant is predicted to be damaging, as reflected by a CADD score ≥ 20 (7).

## Replication of somatic variants

A subset of 44 randomly selected and three biologically interesting (mutations in *STAT3* and *DNMT3A*) somatic variants from 24 individuals were selected for replication (**Supplementary table 2**). The variants were grouped in five pools (7-10 variants from 3-5 individuals per pool). For each pool, a multiplex PCR was optimized and used with DNA extracted from CD4^+^ or CD8^+^ T lymphocytes of each individual in the pool. The PCR mix contained 1x Phusion Master Mix (Thermo Scientific), 0.5 µM of each primer and 140 ng DNA. Initial denaturation took place at 98°C during 30 seconds, followed by 33 cycles of denaturation at 98°C during 10 seconds, annealing at 64°C during 30 seconds and extension at 72°C during 30 seconds and by final extension at 72°C during 5 minutes. Multiplex reactions were cleaned up with SPRIselect (1.0x, Beckman Coulter). Multiplex reactions containing non-overlapping amplicons were pooled, resulting in 10 sequencing pools with 41 or 31 amplicons each. Libraries were prepared using the Nextera workflow (Illumina) and sequenced with the MiSeq PE100 technology (CeGaT GmbH). Pools were demultiplexed using Illumina’s bcl2fastq (version 2.20) and adapters were trimmed with Skewer (version 0.2.2). Sequence reads were aligned to the human Reference Genome Build (NCBI37/hg19) by BWA software (version 0.7.17). Local realignment around indels and base recalibration were done using GATK (version 3.5). SAMtools (version 1.9) was first used to create pileup files per sequencing pool. When running mpileup, we disabled maximum depth and base quality adjustments. VarScan2 (version 2.3.9) was then used for a direct comparison of target and reference pools, with minimal variant frequency set to 0 (3). Primer design was unsuccessful for six randomly selected variants and amplicon sequencing failed for the CD8^+^ subset of one individual (corresponding to eight randomly selected variants), leaving 33 variants from 23 individuals that could be evaluated. Variants were considered as replicated if (i) the correct alternate allele was identified, (ii) the VAF in the target sample is higher than the VAF in the reference sample and (iii) a significant somatic p value is reported (< 0.0001, accounting for multiple testing corresponding to the number of variants tested).

## Characterization of somatic variants

We tested sets of genes affected by somatic variants for over and underrepresentation of (GO) terms related to biological processes using PANTHER (8). Genes affected by somatic variants were grouped all together, or grouped by cell type or disease status (**Supplementary table 3**). The reference gene list was created from the WES target list. GO terms are reported if a false discovery rate (FDR) lower than 5% was obtained with a binomial test.

Mutational signatures were identified with deconstructSigs version 1.8.0 (9) with exome2genome normalization and Single Base Substitution (SBS) mutational signatures v3.2 from March 2021, downloaded from COSMIC (<https://cancer.sanger.ac.uk/signatures/sbs/>), and the SBSblood signature (10). All identified variants were included, grouped by disease status and cell type.

Cell type-specific gene expression was evaluated using normalized expression values from the RNA HPA blood cell gene data, based on The Human Protein Atlas version 20.1 and downloaded from <http://www.proteinatlas.org> (11). We defined “other genes” as all genes from the whole exome panel minus the genes affected by somatic variants in the subset under investigation.

# Analysis of age-matched subset


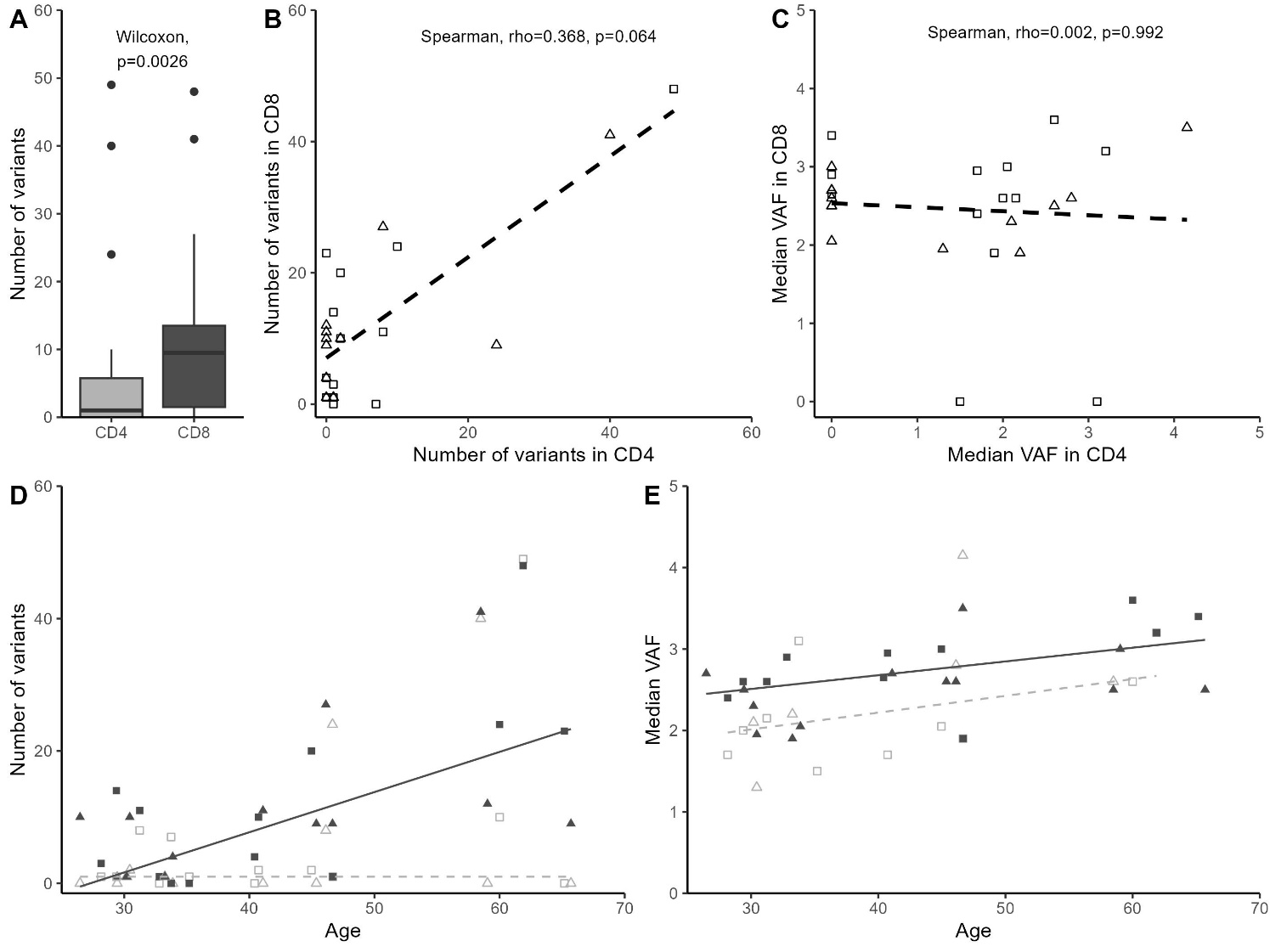


**Supplementary figure 5. Somatic variants are more common in CD8^+^ than in CD4^+^ T lymphocytes and are correlated with the individual’s age in the age-matched subset.**

Data points from controls are depicted by squares, and from persons with MS by triangles. In D, E, data points from the CD4^+^ subset are depicted in light grey and without fill, and from the CD8^+^ subset in solid dark grey. Linear regression trend lines are shown. **A**. Distribution of number of somatic variants in CD4^+^ and CD8^+^ subsets. **B**. Correlation between number of somatic variants in CD4^+^ and CD8^+^ subsets (Spearman rho=0.368, p=0.064). **C**. Correlation between median VAF of somatic variants in CD4^+^ and CD8^+^ subsets, with median VAF set to 0 for subsets without somatic variants (Spearman rho=0.002, p=0.992). **D**. Number of variants in CD4^+^ and CD8^+^ T lymphocytes in function of the individual’s age (median regression model: number of variants increases with age in the CD8^+^ subset (p=0.011), but not in the CD4^+^ subset (p=1)). **E**. Median VAF of variants in CD4^+^ and CD8^+^ T lymphocytes in function of the individual’s age (median regression model: median VAF estimated to be 0.46% higher in CD8^+^ subset (p=0.038) and to increase with 0.02% per one year increase in age (p=0.045)).


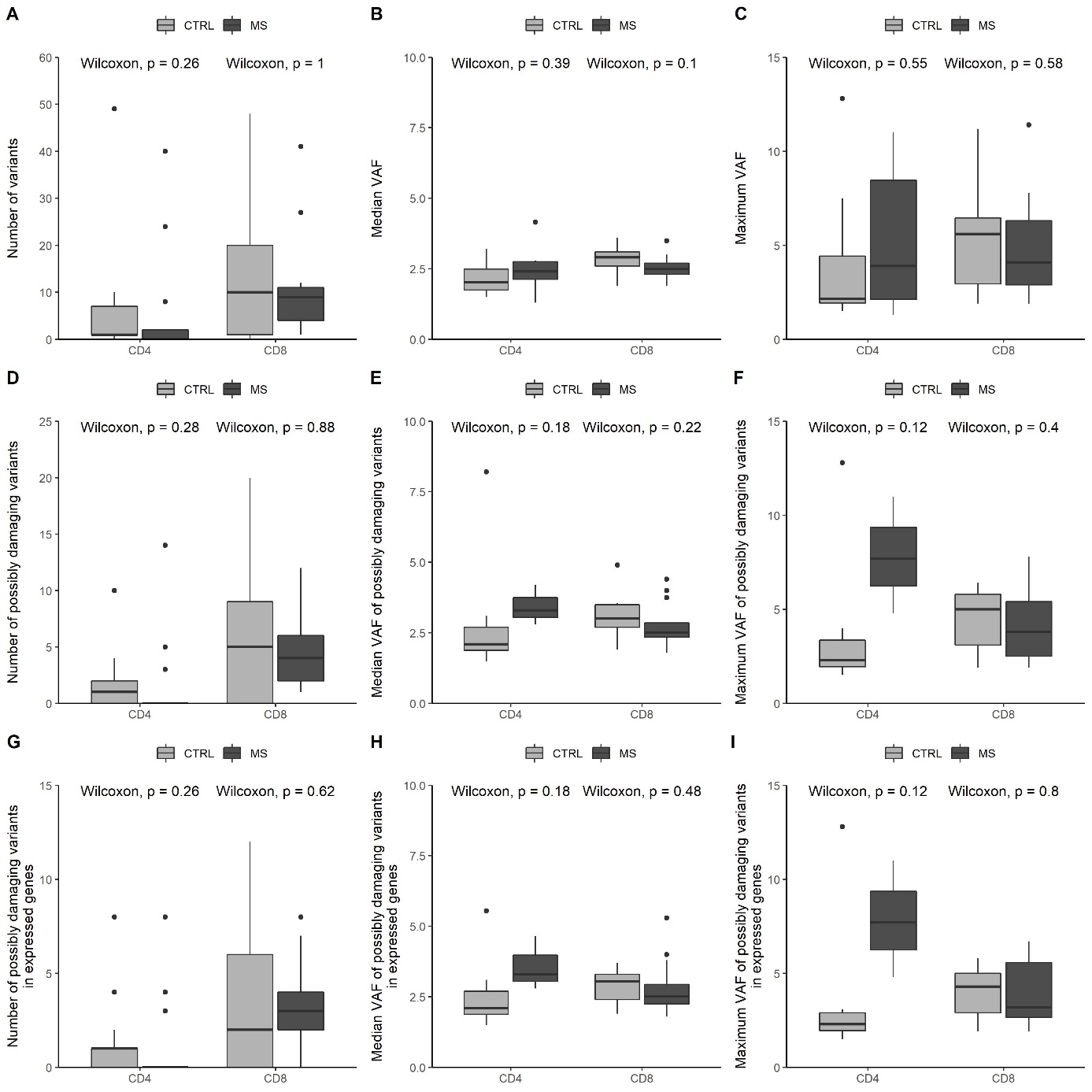


**Supplementary figure 6. Somatic variants are similar in number and abundance in the age-matched control individuals and MS patients.**

**A-C**. Number (**A**), median VAF (**B**) and maximum VAF (**C**) of somatic variants in CD4^+^ and CD8^+^ T lymphocytes in age-matched control individuals and persons with MS. **D-F**. Number (**D**), median VAF (**E**) and maximum VAF (**F**) of somatic variants predicted to be damaging in CD4^+^ and CD8^+^ T lymphocytes in age-matched control individuals and persons with MS. **G-I**. Number (**G**), median VAF (**H**) and maximum VAF (**I**) of somatic variants predicted to be damaging and expressed in the affected cell subset in CD4^+^ and CD8^+^ T lymphocytes in age-matched control individuals and persons with MS.

**Supplementary table 6. Study participant characteristics of age-matched subset.**

|  | **Age‑matched CTRL**  **(N=13)** | **Age‑matched MS**  **(N=13)** |  |
| --- | --- | --- | --- |
| **Gender** |  |  |  |
| Female | 7 (53.8%) | 11 (84.6%) |  |
| Male | 6 (46.2%) | 2 (15.4%) |  |
| **Age** |  |  |  |
| Mean (SD) | 42.3 (12.8) | 42.0 (12.9) |  |
| Median [Min, Max] | 40.4 [28.2, 65.2] | 41.1 [26.5, 65.7] |  |
| **Age at onset** |  |  |  |
| Mean (SD) | - | 39.6 (12.4) |  |
| Median [Min, Max] | - | 38.6 [26.0, 61.9] |  |
| **Disease duration** |  |  |  |
| Mean (SD) | - | 2.40 (1.70) |  |
| Median [Min, Max] | - | 2.50 [0.310, 4.88] |  |
| **Disease course** |  |  |  |
| PPMS | - | 1 (7.7%) |  |
| RRMS | - | 10 (76.9%) |  |
| Unknown | - | 2 (15.4%) |  |
| **Smoking status** |  |  |  |
| Previous smoker | 2 (15.4%) | 4 (30.8%) |  |
| Current smoker | 1 (7.7%) | 1 (7.7%) |  |
| Never smoker | 10 (76.9%) | 8 (61.5%) |  |
| Unknown | 0 (0%) | 0 (0%) |  |

**Supplementary table 7. Sequencing depth of samples of age matched subset.**

|  | **Age‑matched CTRL**  **(N=13)** | **Age‑matched MS**  **(N=13)** |
| --- | --- | --- |
| **Median coverage across exome in CD4^+^ subset** | | |
| Mean (SD) | 364 (50.1) | 376 (48.2) |
| Median [Min, Max] | 372 [218, 410] | 378 [283, 451] |
| **Median coverage across exome in CD8^+^ subset** | | |
| Mean (SD) | 330 (49.8) | 326 (53.1) |
| Median [Min, Max] | 326 [260, 421] | 323 [222, 426] |
| **Percentage of exome with ≥150x in both CD4^+^ and CD8^+^ subset** | |  |
| Mean (SD) | 95.1 (4.15) | 95.5 (4.04) |
| Median [Min, Max] | 97.0 [83.4, 98.4] | 96.6 [83.8, 98.6] |

# Supplementary References

1. van Horebeek L, Hilven K, Mallants K, van Nieuwenhuijze A, Kelkka T, Savola P, et al. A robust pipeline with high replication rate for detection of somatic variants in the adaptive immune system as a source of common genetic variation in autoimmune disease. Hum Mol Genet. 2019;28(8):1369–80.

2. Cibulskis K, Lawrence MS, Carter SL, Sivachenko A, Jaffe D, Sougnez C, et al. Sensitive detection of somatic point mutations in impure and heterogeneous cancer samples. Nat Biotechnol. 2013 Mar;31(3):213–9.

3. Koboldt DC, Zhang Q, Larson DE, Shen D, McLellan MD, Lin L, et al. VarScan 2: somatic mutation and copy number alteration discovery in cancer by exome sequencing. Genome Res. 2012 Mar;22(3):568–76.

4. Jonsson H, Magnusdottir E, Eggertsson HP, Stefansson OA, Arnadottir GA, Eiriksson O, et al. Differences between germline genomes of monozygotic twins. Nat Genet. 2021 Jan 1;53(1):27–34.

5. Wang K, Li M, Hakonarson H. ANNOVAR: functional annotation of genetic variants from high-throughput sequencing data. Nucleic Acids Res. 2010 Jul 3;38(16).

6. Itan Y, Shang L, Boisson B, Patin E, Bolze A, Moncada-Vélez M, et al. The human gene damage index as a gene-level approach to prioritizing exome variants. Proc Natl Acad Sci U S A. 2015 Nov 3;112(44):13615–20.

7. Kircher M, Witten DM, Jain P, O’roak BJ, Cooper GM, Shendure J. A general framework for estimating the relative pathogenicity of human genetic variants. Nat Genet. 2014;46(3):310–5.

8. Mi H, Ebert D, Muruganujan A, Mills C, Albou LP, Mushayamaha T, et al. PANTHER version 16: a revised family classification, tree-based classification tool, enhancer regions and extensive API. Nucleic Acids Res. 2021 Jan 8;49(D1):D394–403.

9. Rosenthal R, McGranahan N, Herrero J, Taylor BS, Swanton C. DeconstructSigs: delineating mutational processes in single tumors distinguishes DNA repair deficiencies and patterns of carcinoma evolution. Genome Biol. 2016 Feb 22;17(1).

10. Machado HE, Mitchell E, Øbro NF, Kübler K, Davies M, Leongamornlert D, et al. Diverse mutational landscapes in human lymphocytes. Nature. 2022 Aug 25;608(7924):724–32.

11. Uhlén M, Fagerberg L, Hallström BM, Lindskog C, Oksvold P, Mardinoglu A, et al. Proteomics. Tissue-based map of the human proteome. Science. 2015 Jan 23;347(6220).
